# Supplementary material for: Median Arcuate Ligament Compression in Orthotopic Liver Transplantation: Results from a Single-Center Analysis and a European Survey Study
Source: J Clin Med. 2019 Apr 23;8(4):550. doi: 10.3390/jcm8040550 (PMC6518097; doi:10.3390/jcm8040550)
Supplement: Supplementary file 1 [file jcm-08-00550-s001.zip › supplementary - for conversion/Supplementary data 1.docx]

Data S1. Survey questions

1. Which approach do you routinely use in pre-operative imaging of recipient vascular anatomy?

- Colour Doppler ultrasound
- CT-Angiography/multi-detector CT-scan
- Conventional angiography
- Other (please specify)

2. Do you routinely look for potential anatomical compression or kinking of the celiac axis (e.g., Dunbar syndrome, Harjola-Marable syndrome, median arcuate ligament-MAL syndrome) in sagittal reconstruction of the CT-scans?

- No, we do not look for the sagittal reconstruction because the axial slides already deliver satisfactory information
- We only look for sagittal reconstruction if there is a suspected alteration visible on the axial sections
- Yes, we routinely look for the sagittal reconstruction in all cases
- Other (please specify)

3. What is your institutional protocol in cases where you find a suspected anatomical compression of the celiac axis in the pre-operative diagnostics (e.g., Dunbar-syndrome)?

- In case of a suspected MALS we always divide the MAL during transplantation
- If pre-operative colour Doppler ultrasound shows abnormal flow in the hepatic artery we divide the MAL intraoperatively, in case of normal flow we do not routinely divide the MAL
- If intraoperative flowmetry shows reduced flow in the hepatic artery following graft reperfusion we divide the MAL, otherwise not
- We do not divide the MAL on the first occasion but we regularly perform colour Doppler ultrasound post-operatively to look for arterial perfusion of the graft
- In such cases we perform alternative revascularisation techniques e.g. aorto-hepatic approach
- Other (please specify)

4. If you do not routinely divide the MAL in cases of a suspected MAL syndrome what is the main reason behind it?

- Limited success and high recurrence rates of the surgical division
- Controversial significance of MALS
- Potential risk of injury
- Other (please specify)
