# Supplementary material for: Median Arcuate Ligament Compression in Orthotopic Liver Transplantation: Results from a Single-Center Analysis and a European Survey Study
Source: J Clin Med. 2019 Apr 23;8(4):550. doi: 10.3390/jcm8040550 (PMC6518097; doi:10.3390/jcm8040550)
Supplement: Supplementary file 1 [file jcm-08-00550-s001.zip › supplementary - for conversion/Supplementary Table 1.docx]

**Table S1.** Clinical details on patients with extrinsic celiac axis stenosis and revascularisation, early arterial complications needing revision or intervention.

| **No.** | **Intrinsic/Extrinsic Stenosis** | **Type of Arterial Revascularisation** | **MAL Division** | **Early Major Arterial Complications** |
| --- | --- | --- | --- | --- |
| 1 | Extrinsic | Standard branch patch | Yes | No |
| 2 | Extrinsic | Standard branch patch | Yes | No |
| 3 | Extrinsic | Standard branch patch | Yes | No |
| 4 | Extrinsic | Standard branch patch | Yes | No |
| 5 | Extrinsic; manifest occlusion with thrombosis | Aorto-hepatic jump graft | n.a. | Yes;  Pseudoaneurysm at the interposition graft and bleeding |
| 6 | Extrinsic | Anastomosis with recipient accessory right hepatic art. originating from the celiac axis | No,  retrospective diagnosis of MALC | Yes;  Early HAT with revision and thrombectomy + MAL division, ReOLT due to graft failure on POD5 |
| 7 | Extrinsic | Standard branch patch | No,  retrospective diagnosis of MALC | Yes;  Early HAT with revision and thrombectomy + MAL division |
| 8 | Extrinsic | Standard branch patch | Yes | No |
| 9 | Extrinsic | Standard branch patch | Yes | No |
| 10 | Extrinsic | Standard branch patch | Yes | No |
| 11 | Extrinsic | Standard branch patch | Yes | No |
| 12 | Extrinsic | Standard branch patch | Yes | No |
| 13 | Extrinsic | Standard branch patch | Yes | No |
| 14 | Extrinsic | Standard branch patch | Yes | No |
| 15 | Extrinsic | Standard branch patch | Yes | No |
| 16 | Extrinsic | Standard branch patch | Yes | No |
| 17 | Extrinsic | Standard branch patch | Yes | No |
| 18 | Extrinsic | Standard branch patch | Yes | No |
| 19 | Extrinsic | Standard branch patch | Yes | No |
| 20 | Extrinsic | Standard branch patch | Yes | No |
| 21 | Extrinsic | Standard branch patch | Yes | No |
| 22 | Extrinsic | Standard branch patch | Yes | No |
| 23 | Extrinsic | Standard branch patch | Yes | No |
| 24 | Extrinsic | Standard branch patch | Yes | No |
| 25 | Extrinsic | Standard branch patch | Yes | No |
| 26 | Extrinsic; milder stenosis (≈50% luminal reduction) | Standard branch patch | No, intraoperatively good arterial flow | Yes;  liver abscess and reduced arterial perfusion in ceCT on POD40, angiography, coil embolization of the splenic artery, significantly improved flow |
| 27 | Extrinsic; manifest occlusion with thrombosis | Reconstruction to the SMA transposed aberrant right HA due to manifest celiac occlusion | No | No |
| 28 | Extrinsic | Standard branch patch | Yes | No |
| 29 | Extrinsic; milder stenosis (≅50% luminal reduction) | Standard branch patch | No, intraoperatively good arterial flow | No |
| 30 | Extrinsic | Standard branch patch | Yes | No |
| 31 | Extrinsic | Aorto-hepatic jump graft | n.a. | No |
| 32 | Extrinsic; severe stenosis without thrombosis | Aorto-hepatic jump graft | n.a., (hypoplastic celiac axis, no standard revascularisation attempted) | No |
| 33 | Extrinsic | Standard branch patch | Yes | No |
| 34 | Extrinsic | Standard branch patch | Yes | No |

Standard branch patch refers to: standard anastomosis using branch patches of the common hepatic artery/splenic artery patch of the donor and the proper hepatic artery/gastroduodenal artery of the recipient. Aorto-hepatic jump graft refers to: supra-celiac interposition graft. Abbreviations used: MAL, median arcuate ligament; SMA, superior mesenteric artery; POD, postoperative day, CT, computed tomography; HA, hepatic artery.
